# New Insight to Overcome Tumor Resistance: An Overview from Cellular to Clinical Therapies

**DOI:** 10.3390/life11111131

**Published:** 2021-10-24

**Authors:** Giulia Mitola, Paolo Falvo, Francesco Bertolini

**Affiliations:** Laboratory of Hematology-Oncology, IEO European Institute of Oncology IRCCS, 16, 20139 Milan, Italy; Giulia.Mitola@ieo.it

**Keywords:** tumor resistance, in vitro and in vivo studies, preclinical studies

## Abstract

Disease relapse caused by drug resistance still represents a major clinical hurdle in cancer treatments. Tumor cells may take advantage of different intracellular and genetic systems attenuating the drug effects. Resistant cells or minimal residual disease (MRD) cells have strong clinical relevance, as they might give rise to secondary tumors when the therapy is concluded. Thus, MRDs are crucial therapeutic targets in order to prevent tumor relapse. Therefore, several groups aim at understanding how MRDs are orginated, characterizing their molecular features, and eradicating them. In this review, we will describe MRD from a genetic, evolutionary, and molecular point of view. Moreover, we will focus on the new in vitro, in vivo, preclinical, and clinical studies that aim at eradicating tumor resistance.

## 1. Tumor Resistance: Biological Mechanisms and Clinical Implications

Despite considerable improvements in cancer patient management and treatment in the last two decades, disease relapse still represents a major clinical hurdle, with many commonly diagnosed cancers showing a ten-year survival rate of around 50% [1]. Development of drug resistance is one of the main causes eventually leading to relapse, and this renders tumors very hard to be eradicated, sometimes even untreatable [2]. While in some cancer types, such as glioblastoma [3] or ovarian cancer [4], the majority of patients undergo relapse (around 100% for glioblastoma and 85% for ovarian), for other tumors types, such as estrogen receptor positive breast cancer or prostate cancer, the proportions are sensibly lower (around 10–20%) [5,6]. Nevertheless, these are still disturbing numbers considering that breast and prostate cancers are very common. Thus, an urgent effort to overcome tumor resistance is needed.

Several groups have studied how tumors become resistant to therapies. Different independent studies have shown that these mechanisms arise during first-line treatments (e.g., chemotherapy, radiotherapy). The exposure to cytotoxic agents mainly attacks rapidly dividing cells, blocking their growth. Unfortunately, cancer cells have evolved several mechanisms in order to counteract drug exposure. One of these mechanisms is based on “masking themselves” by decreasing the rate of cell division. These cells are defined as minimal residual disease (MRD) cells. They are capable of slowing down their own cell cycle upon exposure with cytotoxic agents, and reactivating it in the absence of the drug, possibly giving rise to secondary tumors [7]. Since MRDs are crucial in the reactivation of tumors, their removal is fundamental in order to obtain a complete remission.

The process leading to MRD formation is still only partially known. In many cases, drug toxicity only partially affects MRD viability, since these effects are mitigated by lowering the intracellular concentration of the compound. This is achieved either by expelling the drug out of the cells [8] or by decreasing its uptake [9]. Actually, in the 1970s, it was discovered that upregulation of a particular channel protein called P-glycoprotein was correlated with increased resistance to drugs in Chinese hamster ovary (CHO) cell line [10]. A couple of years later, the function of the protein was elucidated by DNA transfer experiments. When DNA from resistant cells was transferred to nonresistant cells, the latter acquired resistant phenotypes, and this correlated with higher P-glycoprotein expression [11]. In 1985, the gene expressing P-glycoprotein was isolated and the protein was completely characterized to be an ATP-energy-dependent pump that expels small protein from the cells [12].

Alternatively, MRD cells counteract the toxic effects of therapies by preventing apoptosis or by activating protecting mechanisms (such as DNA repair systems). Actually, in several works, it was observed that these cells may harbor many mutations in peculiar genes, such as p53 or Bcl2, blocking the apoptotic cascade [13].

Within some tumor types, MRDs show some features of cancer stem cells (CSCs). Similar to normal stem cells (SCs), cells in this population, sometimes referred to as tumor-initiating cells (TICs), possess self-renewal abilities. Thanks to their proliferative capacity, they can fuel tumor population, sustaining its growth. Analogously to MRD cells, CSCs upregulate cell pumps to efflux drug from the cells and have slower cell cycles [14]. Despite these two populations sharing commonalities, the extent of the similarity between MRD and CSCs and whether these populations actually overlap are still open questions. CSC identification was pivotal to study tumor architecture and organization. Similar to their normal counterparts, tumors are well-organized entities (sometimes hierarchically) [15]. This heterogeneity across cancer cells is one major hurdle to effective cancer therapy. As reported in different cancer types, tumor is not a single disease, but is composed by different cells possessing their own distinctive biological features (Table 1).

Although some of the mechanisms by which cancer cells are resistant to therapy have been partially elucidated, little is known about the evolutionary process leading to the emergence of resistant cells. Is resistance shown after the contact with the drugs or are some cells naturally resistant? This question was highly debated and is considered a trait-de-union between tumor and evolutionary biology, specifically to Lamarck’s and Darwin’s mechanisms of evolution [16]. Briefly, the Lamarckian principle claims that an external stress source pushes organisms’ changes, while the Darwinian claims that innate changes are already present independently from the stress sources. In both principles, if the change gives advantages to the population, it is selected, otherwise the character is counter-selected and repressed. These evolutionary principles can be easily translated to tumor biology in the terms of selection of subclones with fitness advantage to the external pressure (Lamarckian principle) or pre-existent resistant clones (Darwinian principle) [1]. Acquired advantage relies on de novo mutation acquisitions, which render tumor cells more prone to surviving in hostile conditions. On the other hand, a predetermined fitness advantage advocates for pre-existent mutations: some cells are naturally resistant, independently of the presence of external insults. In this case, once the cells are subjected to environmental changes, some are either genetically or nongenetically predisposed to survive and are finally selected. Whether adapting or pre-existing alterations are the main driver of cancer cell resistance is still highly debated.

In vitro studies have demonstrated that MRD cells can already exist as small subpopulations before drugs exposure [17,18]. Several independent observations demonstrated evidence for MRD in different tumor types, including melanoma, breast cancer, and colon cancer [17,18,19]. Nevertheless, they generally failed to identify widespread expansion of clonal subpopulations showing relapse-specific genetic alterations in the relapse. This strongly suggests that nongenetic events are the main driver of the drug-resistant phenotype. Interestingly, in-depth analysis of MRD cells and, specifically, of cells with features of MRD in the primary lesions demonstrated their divergence from the bulk of the tumor at the transcriptional level, with upregulation of specific pathways at the single-cell level [20]. These data argue in favor of a model in which transcriptional fluctuations occurring stochastically in the primary tumor could already generate a subpopulation with features of MRD [17]. Although these observations are completely in agreement with a model of pre-existent resistance, it is difficult to rule out the presence of acquired-drug resistance. It is easier that resistance is a complex process which derives from concomitant pre-existent alterations and acquired ones.

## 2. In Vitro Models to Study Tumor Resistance

In vitro tumor models are the first step to evaluate the efficacy and toxicity of anticancer therapies. This excludes drugs with low efficacy or high toxicity from being used in preclinical mouse models (better explained in the next paragraph). In the field of tumor resistance, the main goal of in vitro systems is to evaluate that a single drug or drug combinations completely eradicates tumor. All the in vitro experiments should obviously be followed by in vivo preclinical models in order to finally validate the efficacy in a more complex system.

The models which are normally used are stabilized cell lines or primary cell lines taken directly from tissues (normally human or mice). Both systems are very useful and present pros and cons: using stabilized cell lines is simpler, culture conditions are well reported in the literature. However, during culturing, cells may harbor de novo mutations and chromosome aberrations [21]. Thus, they may acquire genetic differences from primary tumor. For primary tumor cells, it is more complicated to generate cultures, but they mirror better tumor physiology [22]. Moreover, specific therapies should be used for each patient (for example, for given mutations, particular combinations of drugs may be used), favoring the concept of personalized medicine [23].

More recently, 3D culture, which better resembles tumor physiology and architecture, was favored in drug testing. In this culture method, cells are organized in three-dimensional structures, mimicking cell-to-cell interactions and tissue-specific stiffness, oxygen, and nutrient consumptions [24]. However, in several tumor types also, the microenvironment plays a crucial role; several immune and matrix cells may have pro/anticancer functions [25,26,27,28,29].

There are several examples in the literature in which single agents or drug combinations were used in order to completely eradicate tumors. Here, we listed some recent works in which in vitro models were used to evaluate drug efficacy and dose.

In vitro testing is fundamental, also, to repurpose some drugs for cancer treatments. Drug repurposing consists of the use of a preapproved drug outside its original scope of medication for the treatment of another pathology [30]. In the field of oncology, there are several advantages, as it limits the costs for de novo drug productions and accelerates preclinical tests, since toxicity and administration assays are already performed [31]. In our group Talarico and colleagues evaluated the efficacy of Aspirin and Metformin in the treatment of resistant breast cancer models (BC) [32]. It was shown that the combination of Aspirin and Metformin was effective against BC models in inducing apoptosis in vitro, completely eradicating tumors. Moreover, when this study was performed in vivo, they evaluated that, along with BC cancer cell death, it decreases the proliferation of white adipose tissue progenitors, which have a role in BC progression.

Indeed, as discussed in the previous paragraph, P-glycoprotein is an important receptor that may confer the resistance phenotype. Therefore, it is a main target to ensure complete tumor eradications. In several works, different compounds targeting P-glycoprotein were tested in vitro in combination with classic chemotherapy to evaluate their synergy [12]. For example, in P-glycoprotein-overexpressing colon carcinoma models, Yuan and colleagues demonstrated that cinobufagin, a substance taken from Asiatic toads, increases sensitivity towards Doxorubicin [33]. In another work, Chen and colleagues demonstrated that resistant HeLa to Vincristine treatments reduced resistance when treated with Iso-penicillixanthone-A (iso-PXA), isolated from marine-derived fungus [34].

As discussed above, 3D culture has stronger clinical relevance compared to 2D. It has a lower ethical impact and costs less than animal studies, and biobanks can be created using few materials derived from patients’ tissues [35]. Recently, Van de Wetering and colleagues performed a pharmacological screening on resistant colon cancer organoids. Strikingly, they observed that several used strategies were not effective with particular mutations (e.g., p53 mutants were resistant to MDM2 inhibition) and identified a potential treatment for RNF43 mutants [36]. Taken together, these data are a powerful tool in order to move to personalized medicine.

Altogether, these observations clearly show that in vitro testing is the first step to address specific therapeutic treatments. However, all these studies should be followed by in vivo preclinical tests, which will be better discussed in the following paragraph.

## 3. In Vivo Preclinical Model to Overcome Tumor Resistance

The advent of high throughput cancer genomics, proteomics, and metabolomics analysis have revolutionized the diagnosis of cancer patients and extended the knowledge about the potential response to treatment, thanks also to the use of validated biomarkers [37]. This progress in the study of tumors and the development of new anticancer agents has significantly contributed to the improvement of both disease-free survival and quality of life in cancer patients. Although the problem of cancer therapy resistance is complex, several biological variables might lead to treatment becoming refractory, commonly coexisting in cancer, and occurring in a time- and therapy-dependent way [38].

In many cases, the response to treatment may vary over time due to different biological mechanisms, so leading to cancer relapses and recurrence. Acquired drug resistance is a major problem in fully effective cancer treatments, and the lack of initial response reflects some form of intrinsic drug resistance. Predicting the efficacy of anticancer therapy is a crucial aspect for drug development. As a result, several efforts are being made to identify the resistance mechanism and develop innovative drugs that could overcome them [39].

Various experimental preclinical models are used to study the mechanism underlying tumor resistance. Modeling tumor complexity in cellular and animal models can facilitate the prediction of resistance through molecular and functional studies. Moreover, these models are used to analyze the mechanisms of resistance in order to propose new strategies to circumvent it [40]. Thus, as previously mentioned, cellular in vitro preclinical models can give important information about drugs resistance but they cannot, in toto, adequately replicate human drug exposure, making it difficult to make clinically relevant predictions only from these systems.

Therefore, the use of preclinical animal models can be a useful tool to better understand tumor initiation, progression, and predicting the activity of anticancer agents. Thus, in vivo models should recapitulate the biological characteristics of the tumor and of the related tumor microenvironment. There is a plethora of preclinical in vivo models, and the discovery of new models is constantly evolving. Murine cancer models are among the most advanced in vivo preclinical models for studying cancer, as they are able capture the complexity of human tumors.

Thus, these models are the basis of translational research and provide important information for, understanding the pathophysiology of cancer, including the identification of new target molecules; identification of new therapeutic agents; exploring and combining new therapies; and research on intrinsic and acquired resistance mechanisms to cytotoxicity and targeted therapies [41].

Here we recapitulate the main in vivo models (Table 2) [42]:

1. Cell-line-derived tumor xenograft models (CDX), which are obtained by implanting human tumor cell lines in immunodeficient mice. As the cell lines for the generation of xenograft models are derived from human tumors, the effect of new treatment can be relatively easily studied in these settings [43].
2. Patient-derived xenograft models (PDX), obtained through directly implanting tumor-derived materials into immunodeficient animals. This model permitted important information to be obtained about sensitivity to clinical candidate drugs and the generation of potential prediction markers [44].
3. Syngeneic models rely on the transplantation of mouse tumor cells (derived from the same genetic background) in host animals, either subcutaneously or orthotopically. This model allows the use of fully immunocompetent host mice, useful for studying immune system interaction. These are key models for the evaluation of therapeutics with immune involvement [26,29].
4. Genetically engineered mouse models (GEMMs), characterized by genome alteration, are able to show the role of specific genes in tumor development. These models can mimic the histopathological and molecular feature of human counterparts permitting the successful validation of candidate cancer genes and drug targets [45].

Recent works has shown how the use of preclinical animal models is crucial in the study of new possible treatment approaches for human cancer. For instance, Falvo et al. investigated the therapeutic role of checkpoint inhibitors in combination with chemotherapeutic agents in two in vivo models of triple-negative breast cancer and non-Hodgkin lymphoma [29,46]. Triple-negative breast cancer is among the most aggressive and lethal types of breast cancer, and currently available therapies have unsatisfactory impacts on patients’ survival. Precisely, two preclinical immunocompetent models of TNBC were designed to investigate different schedules of treatments. Thus, in vivo studies indicated that the combinatorial therapy of two chemotherapeutic agents (Cyclophosphamide, Vinorelbine) with CIs (anti-PD-1) was more effective than any other combinatorial regimes in terms of local and metastatic tumor growth. This work set important findings for the treatment of triple breast cancer, which remains most of the time untreated, laying the foundations to design possible novel clinical trials.

In another example of studies on how to overcome tumor intrinsic and/or acquired resistance to treatments options, Irie et al. investigated the treatment approaches in HER2-overexpressing breast cancer. The therapeutical strategies for HER2-overexpressing breast cancer are: HER2-targeting antibodies (trastuzumab, pertuzumab) and HER2-directed antibody–drug conjugate (trastuzumab emtansine: T-DM1) [47]. However, these treatments can become ineffective due to acquired resistance and there is a need for alternatives therapies.

Specifically, different in vivo cancer resistance models are used that are refractory to trastuzumab in combination with petuzumab, or to T-DM1. These models permit the recapitulation of tumor responses to treatment options, tumor microenvironment, tumor–host interactions, and tumor resistance. Thanks to the use of these preclinical mouse models, it was demonstrated that TAS0728, which is an irreversible selective HER2 kinase inhibitor, is a potentially useful treatment option for patients with tumors refractory to the common HER2-targeting antibodies [47]. The importance of preclinical research relies on the possibility to mimic resistance in patients harboring the same mutations.

A special case is colorectal cancer, which is one of the leading causes of cancer-related mortality worldwide [48]. In the last decade, the introduction of targeted therapies in clinical practice targeting related pathways, such as VEGF and EGFR, has changed the therapeutic approach to metastatic colorectal cancer. However, despite the introduction in clinical practice of mAbs against EGFR (cetuximab or panitumumab) used in combination with chemotherapy having led to significant therapeutic efficacy in colorectal cancer patients with RAS wild-type tumors, some patients develop resistance to the treatment [49]. The activation of alternative signaling pathways able to bypass the EGFR may be the cause of resistance to the treatment. Napolitano et al. investigated the mechanisms of tumor resistance and how to circumvent it. The activation of genetic alterations in genes involved in the EGFR pathway has been hypothesized to play a key role in resistance to anti-EGFR drugs, including mutation in KRAS, NRAS, B-RAF, and PIK3CA, and loss of PTEN. In vivo experiments with cetuximab-resistant cell lines showed that single treatment with cetuximab had little effect or no effect on tumor growth, and similar results were obtained with regorafenib alone [50]. In contrast, the combined treatment significantly inhibited tumor growth. Indeed, this study showed that combined treatment could also be a potential therapeutic strategy to investigate in the clinical setting.

Therefore, the combination of both in vitro and in vivo preclinical models has the potential to ensure a higher predictability of favorable outcomes with a robust clinical correlation. In conclusion, the role of multiple preclinical in vitro and in vivo models permitted testing, prediction, and discovery of the therapeutical efficacy of different drugs before advancing to clinical testing, so as to obtain a more predictable drug response in humans.

## 4. Clinical Trials to Overcome Tumor Resistance

Clinical trials are fundamental for the development of new therapies. The final path to the development of a new cancer treatment depends on clinical trials, which are the final step in assessing the efficacy of a new cancer treatment approach [51].

Over the past decade, new discoveries in the field have led to a change in the methods of clinical trial conduction. Testing new drugs or combinations of drugs has shifted from empiricism to hypothesis-driven and biomarker-based studies, setting the new era of precision medicine [52]. The study of new biomarkers and biological drivers of cancer are used to set the possibility of new therapeutic opportunities. Specifically, there was a shift from the evaluation of cytotoxic agents to an increasing number of investigations focusing on molecularly targeted agents and immuno-oncology compounds [53].

Unfortunately, despite the great progress made in the last years, compared to other medical fields, oncology has been one of the under-represented areas for the discovery of new agents. Furthermore, cancer research must face a very low rate of participation to clinical trials, almost 3–5% of people [51]. Despite these discouraging facts, some of the latest research has been central to the advancement of the therapeutic approach for some types of cancers. The *Clinical Cancer Advances 2020: Annual Report on Progress Against Cancer* from the American Society of Clinical Oncology reports that the research advanced in the past years [54].

Here, we reported some examples of studies in which a new drug approach has led to overcoming the tumor resistance. One interesting opportunity in tumor treatment is linked to the combination of drugs to improve the therapeutical effect and overcome the resistance.

A study on newly diagnosed metastatic prostate cancer evaluated the combination of different types of drugs that target the androgen pathway. In the ENZAMET trial (Clinical-Trials.gov identifier: NCT02446405) [55], it was shown that combining target androgen suppression improved progression and overall survival over less specific target androgen receptor therapy (first-line therapy). Anyway, prostate cancer can become resistant due to the low remaining amounts of androgens levels. Thus, the use of Enzalutamide (an androgen inhibitor) blocks the androgen levels in a more specific way than the standard hormone therapy. The 3-year overall survival rate in the Enzatulamide group is estimated to be 80%, compared with 72% in the standard treatment group [55].

Non-Hodgkin lymphoma is a disease originating in the lymphatic system. It includes different subtypes depending on the cell of origin of the disease. Patients with B cell lymphoma can become resistant to the first-line treatment with the anti-CD20 monoclonal antibody rituximab, alone or in combination with polychemotherapy [56]. An investigational drug known as 5F9 is able to block CD47, a protein that protects cells from the immune system. Preclinical studies suggest that combining 5F9 with rituximab may give a synergistic effect in tumor treatment. A phase IIb clinical trial (ClinicalTrials.gov identifier: NCT02953509) [57] published in 2018 assessed both the safety and efficacy of different doses of 5F9 in 22 patients with relapsed or resistant DLBCL. The combination showed promising initial efficacy: after a median time of 22 weeks, half of the patients had an objective response (tumor reduction), and 36% had a complete response. The response rate in the indolent follicular lymphoma (70%) was higher than that of more aggressive diffuse large B cell lymphoma (40%). However, further and more extensive studies have to be carried out.

## 5. Conclusions

In this review, we have discussed how genetically, molecularly, and evolutionary tumor cells generate resistance to therapies. The resistant phenotype has strong relevance for cancer treatments, since it may evolve in tumor relapse. Therefore, preventing resistance has stronger benefits in terms of patients’ survival. As shown with several examples out of the abundant literature in the field, several in vitro, in vivo, and clinical studies have been performed and are currently ongoing to better elucidate these mechanisms. In parallel, high-throughput genomic techniques are used to better select patients who can benefit from new single-drug or combinatorial therapies.

## Figures and Tables

**Table 1:** Main features of: minimal residual disease (MRD), cancer stem cells (CSCs), and tumor-initiating cells (TICs).

Minimal Residual Disease (MRD)	Major cause of relapse in cancer patients Small numbers of tumor cells remain after or during the treatment Caused by the activation of protecting mechanism: expelling drug and/or reducing the uptake of drug
Cancer Stem Cells (CSCs)	Self-renewal abilities Fuel tumor population, sustaining their growth Activation of protecting mechanism: expelling drug and/or reducing the uptake of drug Highly tumorigenic and drug-resistant cells
Tumor-Initiating Cells (TICs)	Self-renewal abilities Tumor initiation and proliferative capacity Responsible for: maintenance, progression, recurrence, and metastasis Highly tumorigenic and drug-resistant cells

**Table 2:** Advantages and disadvantages of the main preclinical mouse models (CDX; PDX; syngeneic; and GEMMs model).

	Advantages	Disadvantages
CDX Model	Human-derived cell line allows a more stable setting New possible treatment options can be studied more easily	Use of immunodeficient mice, which lack immune system microenvironment Differences from the original tumor tissue
PDX Model	Provides realistic tumor heterogeneity Permits studies about drug sensitivity and potential prediction markers	Use of immunodeficient mice, which lack immune system microenvironment
Syngeneic Model	Use of fully immunocompetent host mice Permits studies of new therapeutical options involving the immune system	Murine tumor does not always mimic human tumors Limited number of cells lines, certain tumors are under-represented
GEMMs Model	Genomic alteration allows study of the mechanism underlying tumor development Preservation of human stromal and molecular elements Useful to validate candidate cancer genes and drug targets	Not always able to reproduce the genomic complexity of human tumors Low penetrance and very long in vivo drug testing experiments
